# 

**Published:** 1893-06

**Authors:** 


### CONTENTS:

Article I.—Sensitive Dentine. By W. G. Beers. L. D. S. - 49
" II.—A Case of Irregularity. By P. J. Friedrichs. D. D. S. 56
" III.—Homœopathy in Dental Practice. By Jules J. Sarrazie, D. D. S. - - - - - - - - 59
" IV.—Practical Points. By Dr. Henry Barnes. - - 65
" V.—Great Eras in Life. By W. C. Barrett, M. D., D. D. S. 68
" VI.—What Produces Caries? By Dr. Sudduth. - - 80
" VII.—The Phosphor Necrosis. - - - - - - 82
" VIII.—Bacteria. - - - - - - - - - 85

### COMMUNICATED.

Illinois State Dental Society. - - - - - - 88
Northwestern University Dental School. - - - - 88
University of Buffalo, Dental Department. - - - - 89
The Missouri State Dental Association. - - - - - 89

### EDITORIAL.

The Other Fellow's Point of View. - - - - - 90
For Toothache. - - - - - - - - - 91

### MONTHLY SUMMARY.

Obtunding Dentine. - - - - - - - - - 91
The Therapeutics of Terraline. - - - - - - 92
Extract from a Discussion on Anchorage of Gold Fillings. - - 93
The Contraction of Rubber Plates. - - - - - - 94
The Lady with the Horse Mane. - - - - - - - 95
Carelessly Swallowed Saliva Mixed with Cocaine. - - - 95
A Dental Curiosity. - - - - - - - - - - 96
World's Columbian Dental Congress. - - - - 96
Saccharin as an Antiseptic of the Mouth. - - - - - 96

